# Core Components of Effective Home Visiting Programmes and Parenting Interventions Delivered by Nurses and Midwives—A Scoping Review

**DOI:** 10.1111/jan.70494

**Published:** 2026-01-21

**Authors:** Outi Savolainen, Hanna Rouvinen, Margaret M. Barry

**Affiliations:** ^1^ Department of Nursing Science University of Eastern Finland Kuopio Finland; ^2^ Health Promotion Research Center University of Galway Galway Ireland

**Keywords:** core components, home visiting, maternal and child health, mental health promotion, nurse‐led intervention, parenting, scoping review

## Abstract

**Aim:**

To investigate international evidence on home visits and parenting interventions delivered by nurses and midwives and to identify core components, such as intervention content, programme characteristics, contextual factors and implementation elements shared by effective interventions.

**Design:**

Scoping Review.

**Data Sources:**

Nine academic databases and grey literature were searched between June and August 2024 for studies published between 2020 and 2024.

**Methods:**

Screening and data extraction were independently conducted by two reviewers using covidence. The intervention characteristics were described using the TIDieR framework, and the content was analysed thematically.

**Results:**

Of the 3217 screened studies, 23 met the inclusion criteria. The studies employed various designs, including RCTs, quasi‐experimental, cohort, cross‐sectional, register‐based and single‐case experimental studies. Interventions were typically guided by theories of human ecology, attachment and self‐efficacy. Most used structured materials and were delivered via face‐to‐face home visits by trained nurses, starting during pregnancy and continuing for up to 2 years. Visits ranged from weekly to monthly, mainly to family homes. Interventions were often tailored to family needs and cultural contexts. Five core themes emerged: (1) parenting education, (2) maternal and infant health, (3) mental health and psychosocial support, (4) community connections and (5) cultural sensitivity.

**Conclusion:**

Effective interventions should be early, structured and tailored. Integrating parenting education, health, mental well‐being and cultural sensitivity improves outcomes and scalable family care practices.

**Implications for the Profession and/or Patient Care:**

Findings highlight the need for structured training and support for nurses and midwives. Integrating these interventions into routine services, with attention paid to equity and proportionate universalism, can enhance family outcomes.

**Impact:**

This review addressed the lack of clarity regarding what makes nurse‐ or midwife‐led interventions effective. It identified key components that support child and family well‐being and offers guidance for designing scalable, evidence‐based interventions in maternal and child health services.

**Reporting Method:**

The EQUATOR guidelines for PRISMA were met.

**Patient or Public Contribution:**

No patient or public contributions.

## What Does This Paper Contribute to the Wider Global Clinical Community

- Synthesises recent evidence on effective nurse‐ and midwife‐led home visits and parenting interventions.
- Key intervention components linked to improved child and family outcomes such as early, structured and tailored support are also highlighted.
- Informs the development and adaptation of evidence‐based interventions for maternal and child health services.

## 1 Introduction

The prenatal period, childhood and adolescence are the key foundations for the healthy development, growth and well‐being of individuals. Promoting children's mental health requires focusing on the full environment of their relationships, including the family (e.g., parents and other family members), healthcare providers (e.g., public health nurses, midwives and general practitioners) and childhood care and education settings (e.g., nurses and teachers). Supporting parents and caregivers is central to this effort (Jeong et al. 2021).

Mental health promotion activities are delivered on different platforms, such as primary health care, which provides an important setting for the provision of mental health promotion activities during the prenatal and infancy periods and strengthens the mental health of children and families (WHO 2008). Nurses work in primary health care teams to identify community health needs and the most appropriate ways of intervening to improve community members' health. They work with their clients in a variety of community settings, including home visits, to provide information and support to promote their mental health. It is essential to ensure that nurses have the necessary knowledge, skills and resources to promote mental health (Anttila et al. 2020; Savolainen et al. 2021).

## 2 Background

There is robust evidence that early mental health promotion benefits child development, parenting, maternal health and social functioning, while reducing behavioural problems (Jeong et al. 2021; Britto et al. 2017). Parenting is a key target for early interventions due to its protective role in children's mental well‐being (Perrin et al. 2016).

Home visits are evidence‐based programmes in which professionals, such as nurses or paraprofessionals, provide services in families' homes. These programmes typically begin during pregnancy and continue through the child's first 2 years. They may be offered universally to all families or targeted to those in vulnerable situations needing additional support. Home visits may focus on young children, children with special healthcare needs, parent–child relationships or parental well‐being (Duffee et al. 2017).

Parenting programmes aim to strengthen parental skills and capacity, often focusing on emotional regulation, consistency and warmth, which are factors linked to positive child mental health outcomes. These programmes vary in format and target age groups, with many designed for parents of children aged 3 to 10, and can also be delivered by nurses as part of family support services (Jeong et al. 2021).

Nurses play a key role in early childhood services and are well‐positioned to support families through routine health visits. In Finland, public health nurses are central actors in maternal and child health services, providing opportunities to integrate mental health promotion into the existing service structures (Grym and Borgermans 2018). Children and family services aim to implement proportionate universalism but face challenges such as regional and population group disparities in early support and service availability. Insufficient resource allocation to disadvantaged families increases child protection needs and out‐of‐home placement. Mental health services for children, particularly those for vulnerable children, are also lacking. The frequency and comprehensiveness of home visits by public health nurses during pregnancy and in families with children varies across municipalities, leading to unequal support and guidance owing to differing resources and local practices (Eklund Karlsson et al. 2022). Therefore, understanding what is effective in nurse‐delivered interventions is essential for informing both practice and policy.

Systematic reviews and meta‐analyses have provided evidence that home‐visiting programmes are associated with improvements in parents' behaviours and skills, such as increased positive parenting and a reduced likelihood of maltreatment (Casillas et al. 2016), as well as positive child outcomes. These include improvements in child mental health, psychosocial and developmental outcomes, reduced risk of child abuse, better socioemotional and behavioural functioning, enhanced cognitive development and improved parent–child interaction (Cibralic et al. 2025). Additionally, numerous parenting programmes have proven their effectiveness both nationally and internationally, showcasing their large‐scale efficacy. Despite promising results, evidence remains inconsistent, and the specific components driving effectiveness are unclear (Cibralic et al. 2025; Molloy et al. 2021; Beatson et al. 2021).

Further research is needed to identify the characteristics and core components of effective nurse‐delivered home visits and parenting interventions. Core components are essential functions or principles and related activities needed to achieve desired outcomes (Blase and Fixsen 2013). Effective intervention strategies include clearly describing the programme's context, core components and active ingredients. These elements must be operationally defined to ensure that they are taught, learned and implemented in typical settings. Additionally, practical strategies for assessing behaviours and practices that reflect the intervention's values and principles are crucial. Understanding whether the core components are correctly implemented is vital to determining whether an intervention's ineffectiveness is due to poor implementation or inherent flaws (Blase and Fixsen 2013).

Identifying and analysing core components can help strengthen existing programmes and inform the development of training and support for health professionals working with children and families. In addition, the identification of core components can facilitate the planning of alternative methods of operation that are potentially simpler to implement and less expensive than the programme as a whole (Engell et al. 2023). This information is valuable for mental health promotion programme planners, especially in regions where the high costs of full programmes make them infeasible. Information on individual intervention elements that are easier to implement than the entire programme can also be used to train public health nurses and midwives by providing them with practical, easy‐to‐use tools to support their everyday work.

## 3 The Review

### 3.1 Aim

This scoping review aimed to identify the core components of effective home visits and parenting interventions delivered by nurses or midwives. The research questions guiding this review were as follows: What are the core components of nurse‐ or midwife‐led home visits or parenting interventions that have demonstrated positive outcomes for children and parents? Accordingly, the objectives of the review were to (1) identify the characteristics of the interventions, such as the intervention type, participant recruitment and intervention goals and (2) examine the content of the interventions.

### 3.2 Methodology

This review employed a scoping review methodology known for its flexibility and iterative nature. Unlike traditional reviews, scoping reviews address broad topics without strict methodological constraints, allowing for analytical reinterpretation and adaptation to emerging themes. This approach often integrates perspectives of multiple disciplines (Gottlieb et al. 2021). Scoping reviews are particularly valuable for setting research priorities, clarifying concepts and definitions, developing research frameworks and providing background or contextual information regarding various phenomena. They are also effective in identifying knowledge gaps, mapping existing literature and examining research practices (Pollock et al. 2021).

This review adopted the scoping review framework developed by Arksey and O'Malley (2005). This framework is particularly useful for mapping the key concepts underpinning a research area and identifying the main sources and types of evidence available. The framework involves five stages: identifying the research question; identifying relevant studies; article selection; charting the data; and collating, summarising and reporting the results. Each stage was meticulously designed to ensure a thorough and systematic literature review, thereby providing a comprehensive and structured overview of the research landscape. This review was prepared in accordance with the PRISMA ScR (Preferred Reporting Items for Systematic Reviews and Meta‐Analyses extensions for Scoping Reviews).

The Template for Intervention Description and Replication (TIDieR) checklist was used to support consistent and transparent reporting of the characteristics and content of the interventions in this review. The use of TIDieR enhances the clarity and replicability of intervention descriptions, which is essential for applying evidence‐based home‐visiting practices in real‐world healthcare settings and strengthening future programme development and implementation (Hoffmann et al. 2014). The TIDieR framework includes information on the rationale and theoretical framework for the interventions (why), the procedures and materials used (what), the delivery methods (how), the timing and dosage of the interventions (when and how much), the locations where they were conducted (where), the individuals who provided the interventions (who) and any customisation involved (tailoring). These elements were used to identify the characteristics of the intervention. Although the TIDieR framework also includes sections on ‘modifications’ and ‘how well’, these details were not included in this review as they were deemed outside of the scope of this particular study's objectives. The artificial intelligence‐generated content tool Microsoft Copilot was used to improve the spelling, grammar and general editing of the manuscript.

#### 3.2.1 Search Methods

The search strategy focused on locating relevant studies from academic databases and grey literature. First, a preliminary search was conducted in PROSPERO to identify any ongoing reviews on the subject, with an additional search conducted within the Open Science Framework (2025). No existing protocols exist for this topic. The review protocol was prepared by the research team and is available in the Open Science Framework (OSF). An academic librarian was consulted to refine the search strategy and ensure comprehensive coverage. The academic databases selected for the searches included PubMed/MEDLINE, Scopus, EBSCO/CINAHL/PsycINFO/ERIC, Web of Science, Social Science Premium Collection, Medic, JBI Evidence‐Based Practice, Cochrane Library (including the Cochrane Database of Systematic Reviews and Cochrane Central Register of Controlled Trials) and the International Clinical Trials Registry Platform.

Grey literature was identified by searching Google Scholar, grey literature databases (Ethos, ProQuest and Finna) and relevant global and national sources such as WHO, UNICEF, the Ministry of Social Affairs and Health in Finland and the Finnish Institute for Health and Welfare (THL).

##### 3.2.1.1 PCC Framework

This review was based on the PCC (participants/concept/context) framework recommended by the Joanna Briggs Institute (Peters et al. 2020). The search terms were prepared using the PCC framework; the population included children and adolescents aged 0–16 years, parents (including the prenatal period) and families. While home visits and parenting interventions typically focus on the early years of life, this review also aimed to include potential interventions targeting older children, specifically those within the age range of Finnish primary school children. The concept covers evidence‐based home visits and parenting interventions. This study included registered nurses and midwives. The searches were conducted using combinations of search terms from electronic databases with advanced search options, as shown in Table 1. The same search terms were used for grey literature searches in Ethos, ProQuest, Finna and Google Scholar.

**TABLE 1:** PCC and Search Strategy.

PCC	Search strategy
Population: children and adolescents aged 0 to 16 years, parents (including the prenatal period) and families	(child* OR infant OR infancy OR newborn OR “early childhood” OR adolescent* OR parent* OR mother* OR father* OR quardian* OR famil* OR” family with child*” OR prenatal OR “postnatal care” OR maternal)
Concept: evidence‐based home visiting programmes/interventions and parenting programmess/interventions	(“home‐visiting program*” OR “home visiting program*” OR “home visits” OR “home‐visiting intervention*” OR “home visiting intervention*” OR “home visiting” OR “parenting program*” OR “parenting intervention*”)
Context: registered nurses and midwives	(“public health nurse*” OR “health visitor*” OR “family nurse*” OR “healthcare professional*” OR” health care professional*” OR nurse* OR “registered nurse*” OR midwife*)

#### 3.2.2 Eligibility Criteria

In this scoping review, particular interest was given to home visiting and parenting interventions delivered by nurses and midwives that have demonstrated positive effects on children's and parents' mental health and well‐being, the social and emotional development of both children and parents, responsive caregiving, quality of parent–child communication and parenting skills in high‐income countries. This review considered both experimental and quasi‐experimental study designs, including randomised and non‐randomised controlled trials, before‐and‐after studies and interrupted time series studies. Analytical and descriptive observational studies were included. Manual searches of the reference lists of all selected articles were conducted. Peer‐reviewed articles, organisational reports and grey literature (i.e., dissertations) were included in this review.

#### 3.2.3 Inclusion and Exclusion Criteria

The selection of studies for review was rigorously conducted in accordance with the inclusion and exclusion criteria detailed in Table 2. Only studies published in English and Finnish were included. This decision was based on practical considerations but may introduce language bias and limit the generalisability of the findings.

**TABLE 2:** Inclusion and exclusion criteria.

Inclusion criteria	Exclusion criteria	Rationale
**Population**		
Children aged 0 to 16 years. Parents of children aged 0 to 16 years including the prenatal period	Families with children with specific developmental disabilities or chronic illnesses, prematurely born infants or families where one or both parents have a chronic illness, mental health disorder, long‐term mental health problems or substance use disorders	The population spans a wide developmental range, highlighting the impact of parental involvement on child development. Specialised interventions for excluded groups are beyond the scope of this review
**Concept**		
Universal and targeted home‐visiting and parenting interventions which have demonstrated positive effects on: (1) children's mental health or well‐being, (2) parents' mental health or well‐being, (3) social and emotional development of child or parent, (4) responsive caregiving, (5) quality of parent–child communication or (6) parenting skills	Studies focused solely on palliative care, obesity prevention, oral health, infant sleep or breastfeeding Cost‐effectiveness studies Digital interventions	The comprehensive approach captures a wide range of beneficial outcomes, while excluded topics require specialised interventions beyond the review's scope. The focus was on the direct impact of in‐person programmes and interventions on the specified outcomes aiming to ensure consistency in delivery mode and comparability of core components
**Context**		
Predominantly delivered by registered nurses or midwives (over 50% contribution)	Studies conducted solely in low‐ or middle‐income countries Studies conducted in a prison context	Aligns with aim, research question and objectives. The focus was on generalisable and relevant contexts
**Study characteristics**		
Primary peer‐reviewed studies Published from 2020 to 2024 Conducted in high‐income countries Published in English or Finnish	Study protocols Reviews, qualitative studies, mixed method studies including only qualitative results Studies with statistically non‐significant results	Focusing on accessible languages and relevant contexts aligns with the scope of the review. Excluding certain study types maintains the focus on robust, quantitative evidence and supports the synthesis of comparable, measurable outcomes necessary for identifying core components

#### 3.2.4 Study Selection

**FIGURE 1:**
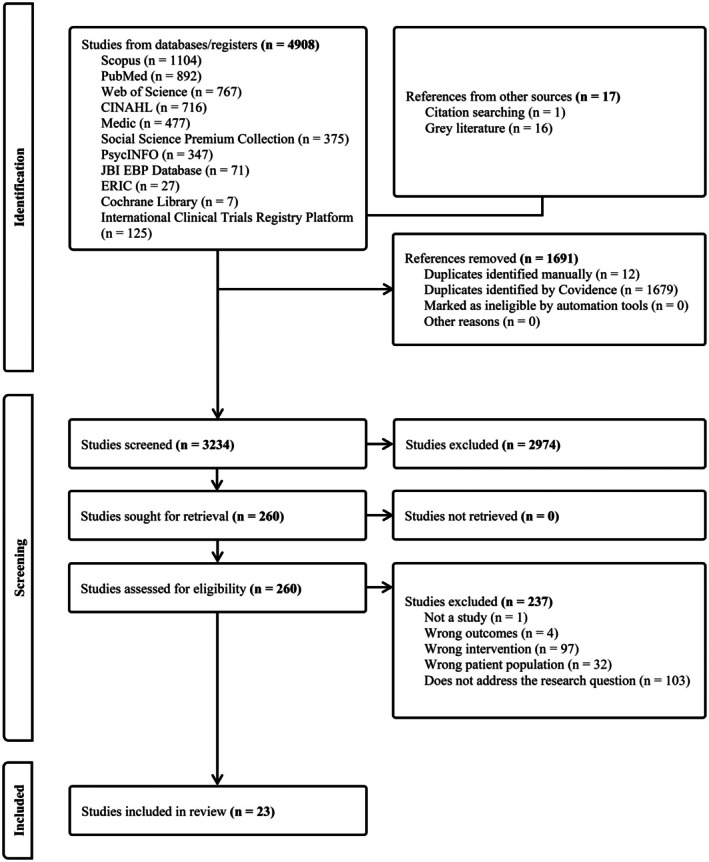
PRISMA ScR flowchart for scoping review (Page et al. 2021; Tricco et al. 2018).

The initial database and grey literature searches yielded 4908 potential studies. The titles and abstracts were imported into Covidence. After removing duplicates (*n* = 1691), two reviewers screened the titles and abstracts of 3234 studies, resolving conflicts and reviewing selections. The original time frame was from 2014 to 2024. During screening, we decided to tighten the timeframe to include studies from 2020 to 2024, and studies from 2014 to 2019 were excluded. The rationale for this restriction was that articles reported before 2020 included those conducted before the 2000s, and the aim was to include the most recent research data. This left 260 studies for a full‐text review. During the full‐text screening, two reviewers independently screened all articles for eligibility and established interrater reliability. Conflicts were resolved, and the findings were discussed by the research team. The screening process followed the best practices for scoping reviews (Page et al. 2021) and is illustrated in the PRISMA‐ScR Flow Diagram (Figure 1).

#### 3.2.5 Data Charting and Extraction

One reviewer (the first author) initially extracted and summarised the data. The process was then reviewed and verified by the co‐authors to ensure accuracy and consistency. A descriptive analytical method was used to extract information from the included articles (Arksey and O'Malley 2005). Articles were categorised according to author information, study aim, design, sample, context and main findings (Table 3). Traditionally, quality appraisal of research is not part of the scoping review process (Munn et al. 2018) and thus was not conducted on the selected articles.

**TABLE 3:** Studies on effective nurse‐led home‐visiting and parenting programmes.

Author(s), year and country	Study purpose	Data/Sample	Method	Main findings
**Universal home visiting programmes/interventions**				
Baziyants et al. (2023) US	To determine sustained (2‐year) impact of random assignment to FC on parenting behaviour and parent mental health and identify heterogeneity of effects	Families of infants *n* = 496 (treatment *n* = 255, control *n* = 241)	RCT	Mothers assigned to FC engaged in more self‐reported positive parenting relative to control mothers (*B* = 0.21; *p* < 0.05)
Kristensen et al. (2020) Denmark	To investigate the effect of health visitors' use of the NBO system in new families	Families *n* = 2566 (intervention *n* = 1332, comparison *n* = 1234)	RCT	Mothers in the intervention group reported higher levels of knowledge about infant communication, responsiveness to cues and soothing techniques compared to the comparison group (*p* < 0.05); small, non‐significant improvements were also observed in maternal confidence and mood, infant socio‐emotional behaviour and the parent‐infant relationship
Goodman et al. (2021) US	To determine the effect of randomisation to FC on child maltreatment investigations and emergency medical care through 5 years of age	Families *n* = 4777 (intervention *n* = 2327, services as usual *n* = 2440)	RCT	Families in the intervention group had fewer CPS investigations (39%) and less child emergency medical care use (33%) compared to controls (90% CI for both outcomes excluded zero); effects were consistent across subgroups, with larger effects for non‐minority families
**Targeted home visiting programs/interventions**				
Catherine et al. (2020) Canada	To evaluate NFP's effectiveness in improving child and maternal health (impact on prenatal cigarette, alcohol, cannabis and use of street drugs)	Pregnant girls and women aged 14–24 years who were preparing to parent for the first time *n* = 739 (intervention *n* = 368, comparison *n* = 371)	RCT	Mothers in the intervention group reported lower cigarette use (−1.6 cigarettes in 2 days, 95% CI: −6.4 to −1.3) and reduced prenatal cannabis use (−6.4 percentage points, 95% CI: −17.0 to −1.7) compared to controls
Catherine et al. (2024) Canada	To evaluate NFP's effectiveness in improving child and maternal outcomes by child age 2 years compared with existing services in British Columbia	Pregnant girls and women aged 14–24 years who were preparing to parent for the first time *n* = 739 (intervention *n* = 368, comparison *n* = 371)	RCT	Mothers in the intervention group reported higher child language scores (+31.3, 95% CI: 0.96 to 61.7) and lower problem‐behaviour scores (−2.2, 95% CI: −3.6 to −0.8) compared to the comparison group
Cavallaro et al. (2024) UK	To determine the effect of FNP on maternal and child outcomes, including identifying which families benefit the most from FNP	Mothers aged 13–19 *n* = 25,680	Cohort study	Children in the intervention group were slightly more likely to achieve a Good Level of Development at age five (RR 1.05, 95% CI: 1.00 to 1.09), and mothers were slightly less likely to have a subsequent birth within 18 months (RR 0.92, 95% CI: 0.88 to 0.97), compared to the comparison group
Chin et al. (2023) Sweden	To evaluate the effect on the health of the child and the mother when the extended home visiting programme is supplemented by a home visit by a midwife and a parent advisor at the end of the pregnancy	Firstborn children *n* = 85 (study *n* = 30, control *n* = 55)	Cross‐sectional study and register study, pilot	Mothers in the intervention group were more likely to undergo postnatal depression screening (90% vs. 65%) and use contraception (50% vs. 34%) compared to controls. Breastfeeding rates were higher at the first home visit (90% vs. 67%) and at 6 months (63% vs. 47%). All children in the intervention group were vaccinated according to the national schedule (vs. 4% unvaccinated in controls). However, a higher proportion of intervention families had at least one smoking parent (63% vs. 23%)
Conti et al. (2021) Germany	To examine the effectiveness of the home visiting programme Pro‐Kind.	Mother–child dyads *n* = 109	RCT	Girls in the intervention group showed more positive mother–child interactions: they were 15 percentage points more likely to show orientation and 3.4 percentage points less likely to show negative or lacking contingency compared to controls; no significant effects were observed for boys.
Goldfeld et al. (2021) Australia	To examine the effects of an Australian nurse home visiting programme right@home offered to pregnant women experiencing adversity, on maternal mental health and well‐being at child aged 3 years	Pregnant women *n* = 722 (intervention *n* = 255 of 363 (70%), control *n* = 240 of 359 (67%))	RCT	Mothers in the intervention group reported better mental health compared to controls, with small effect sizes for reduced depression (*d* = 0.25), anxiety (*d* = 0.20) and stress (*d* = 0.17) and improved total mental health (*d* = 0.23), personal well‐being (*d* = 0.16, 95% CI: 0.04 to 0.29) and self‐efficacy (OR = 1.60, 95% CI: 1.19 to 2.16)
Goldfeld et al. (2022) Australia	To evaluate the benefits of an Australian nurse home visiting programme right@home in promoting children's language and learning, general and mental health, maternal mental health and wellbeing, parenting and family relationships, at child ages 4 and 5 years	Pregnant women *n* = 722 (intervention *n* = 255 of 363 (70%), control *n* = 240 of 359 (67%))	RCT	At 5 years, children in the intervention group showed improved mental health (lower SDQ Externalising and Total Scores), better language and phonological awareness (CELF and SEAPART) and improved self‐control (SSIS). Parents reported benefits in parenting (greater warmth, reduced hostility and conflict, improved efficacy and more regular routines), and mothers reported better mental health and wellbeing (reduced stress, improved personal wellbeing, quality of life and self‐efficacy), as well as less emotional abuse from a partner. One outcome favoured the control group: fewer intervention mothers reported studying at 5 years
Kanda et al. (2022) Australia	To examine the relationship between customised care related to the mother's risk factors and parent satisfaction and enablement in the delivery of a MECSH‐based programme	Pregnant women *n* = 722 (intervention *n* = 352, control *n* = 370)	Cross‐sectional study	Mothers who received appropriate customised care for mental health risk reported significantly greater satisfaction with care compared to those who did not receive such care
Kliem and Sandner (2021) Germany	To examine the effectiveness of a home visiting programme at primary school age	Pregnant women *n* = 755 (intervention *n* = 394, control *n* = 361). Assessments were completed on 533 seven‐year‐old firstborn offspring (intervention *n* = 275, control *n* = 258)	RCT	Mothers in the intervention group reported fewer child behavioural problems (ES = 0.21, 95% CI: 0.03 to 0.38), less abusive parenting (ES = 0.19, 95% CI: 0.00 to 0.37), fewer maternal mental health problems (ES = 0.25, 95% CI: 0.07 to 0.43) and higher maternal life satisfaction (ES = 0.25, 95% CI: 0.07 to 0.43); effects were larger for boys and their mothers
Nguyen et al. (2024) Australia	To investigate the complementary impact of NHV from pregnancy to child age 2 years and quality early childhood education and care from 3 to 4 years on children's developmental outcomes at 4 years (language, executive functioning, behaviour and wellbeing)	Pregnant women *n* = 722 (intervention *n* = 363, control *n* = 359)	RCT and cohort	Children receiving both NHV and quality ECEC showed higher language scores (CELF core language MD = 6.01, 95% CI: 0.1 to 12.0; word structure MD = 1.35, 95% CI: 0.3 to 2.5) and better executive functioning (NIH card sort MD = 6.45, 95% CI: 1.5 to 11.4) compared to those receiving neither. Children with NHV only or ECEC only had lower internalising behaviour scores (SDQ MD = −0.55, 95% CI: −1.0 to −0.1) and better socio‐emotional scores (PedsQL MD = 2.59, 95% CI: −0.1 to 5.3) than those with neither. NHV only was associated with lower internalising scores (MD = −0.51, 95% CI: −0.9 to −0.1), and children receiving both showed a trend toward better executive functioning (MD = 4.59, 95% CI: −0.8 to 10.0, *p* = 0.09)
Price et al. (2023) Australia	To investigate the effects of the Australian right@home nurse home‐visiting programme when children turned 6 years old and started school	Pregnant women experiencing adversity *n* = 722 (right@home programme *n* = 363, usual care *n* = 359)	RCT	Four years post‐intervention, the programme group showed benefits in teacher‐reported executive function (planning ES = 0.23; regulation ES = 0.15) and reading simple sentences (OR = 1.65), as well as parent‐reported behavioural outcomes (SDQ total ES = 0.16; SSIS ES = 0.19). Parenting and family relationship measures favoured the intervention, with increased parental warmth (ES = 0.26), reduced child–parent conflict (ES = 0.26) and improved maternal well‐being (ES = 0.24). Mothers in the intervention group reported less emotional abuse (OR = 1.82), higher self‐efficacy (OR = 1.45) and more plans for future study (OR = 1.28) compared to controls
Robling et al. (2022) UK	To measure effectiveness of FNP home‐visiting programme in reducing maltreatment and improving maternal health and child health, developmental and educational outcomes; explore effect moderators, mediators; describe costs	Mothers aged 19 years or younger *n* = 1618 and their firstborn child(ren) who had participated in the BB: 0–2 trial	Cohort study	Children in the intervention group were more likely to achieve a good level of development at school entry (adjusted OR = 1.24, 95% CI: 1.01 to 1.52) and to meet expected reading standards at KS1 (adjusted OR = 1.26, 95% CI 1.02 to 1.57) compared to controls
Ruiz‐Zaldibar et al. (2021) Spain	To assess the effects of a parenting programme on parental self‐efficacy and parenting styles	Parents *n* = 25 (intervention *n* = 15, control *n* = 10)	RCT, pilot	The programme showed moderate short‐term benefits in parental self‐efficacy (ηp^2^ = 0.095) and some parenting dimensions (emotion, empathy, discipline, self‐acceptance), but these effects were not maintained at follow‐up; a similar trend was observed for parenting styles
Slade et al. (2020) US	To test four main hypotheses, predicting that as compared to dyads in the control condition, (a) MTB mothers would have higher parental reflective functioning; (b) dyads participating in MTB would be less likely to have disrupted affective communication; (c) MTB infants would be more likely to be securely attached, and less likely to be disorganised; and (d) the MTB intervention would have a positive impact on maternal depression and posttraumatic stress disorder	Mothers *n* = 156 (intervention *n* = 77, control *n* = 79)	RCT	Mothers in the intervention group were 2.15 times more likely to be in a higher Reflective Functioning (RF) category than controls (OR = 2.15, 95% CI: 1.02 to 4.53). At 12–14 months, infants in the intervention group were more likely to have organised attachment (OR = 2.69) and secure attachment (OR = 2.59) compared to controls. Strong positive associations were found between baseline and 24‐month measures of parental RF (OR = 15.8), depressive symptoms (OR = 6.11) and PTSD symptoms (OR = 3.13–5.61)
van der Stouwe et al. (2023) the Netherlands	To examine the effectiveness of the added intervention components	Mothers *n* = 9	Single‐case experimental study	There was a significant reduction in parental anger during the T + F phase compared to baseline (B), with a non‐significant increase in anger at baseline (*β* = 0.01, *p* = 0.257) and a significant decrease during T + F (*β* = −0.02, *p* = 0.044)
Mekhail et al. (2023) Sweden	To gain knowledge about the impact of an extended postnatal home visiting programme on parents' comprehensive health literacy (CHL) in multicultural, socioeconomically disadvantaged Swedish settings	First‐time parents, first interview *n* = 193, second interview *n* = 151	Quasi‐experimental study	Parents needing language interpreters in the intervention group showed significant improvement in CHL from pre‐ to post‐measures (*F* = 11.429, *p* < 0.001), with greater gains compared to a similar subgroup receiving only the standard Child Health Care programme (*F* = 5.025, *p* = 0.027)
Waters et al. (2022) UK	To assess the effectiveness of FNP in improving the language outcomes of first‐time teenage mothers and their infants	Pregnant young mothers *n* = 476 (FNP plus usual care *n* = 243, usual care alone *n* = 233)	RCT	Mothers in the FNP group showed a small but significant increase in productive language, with higher mean length of utterances compared to usual care (mean difference = 0.10, *p* < 0.05, 95% CI: 0.004 to 0.20, *d* = 0.18)
**Universal parenting programmes/interventions**				
Sourander et al. (2021) Finland	To investigate the benefits reported by first‐time parents after attending a Families First mentalisation‐based group intervention and look for indicators of mentalisation	Parents *n* = 550 (mothers *n* = 367, fathers *n* = 183)	Cross‐sectional study, pilot	Parents reported peer support as the main benefit from the parent group (39.5% of mothers' codes, 30% of fathers' codes). Understanding their baby was especially valued by fathers (32.7% of fathers' codes) and also by mothers (17.1%). Additionally, 24.8% of mothers' and 1.1% of fathers' codes related to deeper understanding of their own behaviour and relationship with their baby. Benefits regarding family and partner relationships were reported by 6.6% of mothers' and 5.4% of fathers' codes
Tauriello et al. (2023) US	To examine effects of the INSIGHT study responsive parenting intervention on reported and observed general parenting and child behaviour during early and middle childhood	Primiparous mother‐newborn dyads *n* = 279	RCT	RP group children had fewer mother‐reported externalising (*F* = 8.69, *p* = 0.004) and problem behaviours at age three (*F* = 4.53, *p* = 0.03), and showed higher prosocial (*F* = 4.73, *p* = 0.03) and lower antisocial behaviour (*F* = 4.79, *p* = 0.03) at age six compared to controls. At age 6, RP group mothers reported greater use of structure through consistent rules and routines (*F* = 5.45, *p* = 0.02) and better organisation of the child's environment (*F* = 7.12, *p* = 0.008) than controls
**Targeted parenting programmes/interventions**				
O'Farrelly et al. (2021) UK	To test the effectiveness of a brief parenting intervention, the VIPP‐SD, in preventing behaviour problems in at‐risk children aged 12 to 36 months as compared with usual care	Caregivers of children *n* = 300 (VIPP‐SD *n* = 151, usual care *n* = 149)	RCT	The VIPP‐SD group showed fewer behaviour problems than the control group, with a mean difference in total Preschool Parental Account of Children's Symptoms score of 2.03 (95% CI: 0.06–4.01, *p* = 0.04, *d* = 0.20), particularly in conduct symptoms (mean difference 1.61, 95% CI: 0.44–2.78, *p* = 0.007, *d* = 0.30)

#### 3.2.6 Data Analysis

The characteristics of the interventions, such as its type, participant recruitment and goals, were described using the TIDieR framework (Hoffmann et al. 2014). Table 4 briefly describes the characteristics of the programmes and interventions according to the TIDieR framework. A more detailed description of these characteristics is presented in Table S1.

**TABLE 4:** Characteristics of interventions—TIDieR framework (Hoffmann et al. 2014).

Programs/interventions	Family connects Nurse‐Family Partnership Family Nurse Partnership Extended postnatal home visiting program 1 Pro‐Kind right@home Newborn Behavioural Observations Video‐feedback Intervention PUEDES Infancy program Minding the Baby Families First VoorZorg INSIGHT Extended postnatal home visiting program 2
**Why** Rationale	Child health and development Parenting skills and practices Pregnancy and birth outcomes Family support and resources Prevention of child maltreatment Economic self‐sufficiency Mental health and well‐being Equality and participation
Theoretical framework	Human Ecology Theory Attachment Theory Self‐Efficacy Theory Personal Construct Theory Theories of Helper Characteristics and Helping Processes Synactive Theory of Development Social Learning Theory Mentalisation Framework
**What** Procedures and materials used	Recruitment strategies without strict eligibility criteria (e.g., open enrollment) Tailored support for first‐time parents Interventions targeting socially and economically disadvantaged families Assessment and addressing of specific risk factors Use of standardised guidelines and structured protocols for assessment and intervention delivery
**How** Delivery methods	Face‐to‐face home visits to families Face‐to‐face home visits to families and a final telephone contact Face‐to‐face group meetings (the location not mentioned) Observation‐based parent feedback sessions Motivational interviewing techniques and individualised intervention plans Group sessions and peer support activities
**When and how much** Timing and dosage of the interventions	Early start: pregnancy or first weeks Regular visits: few to 50+, weekly to monthly Extended duration: up to 2 years Comprehensive approach addressing health, development and social support
**Where** Locations	Child's home Other calm and child‐friendly environments
**Who** Individuals who provided the interventions	Qualified nurses Multidisciplinary teams Specialised training Trained group leaders
**Tailoring**	Personalised support Cultural adaptation Flexible scheduling Group and individual support

Additionally, the content of the interventions was examined through thematic analysis, focusing on core components. The steps of thematic analysis were as follows: First, the content of the interventions was carefully examined. Next, relevant segments including sentences and text passages describing the content were identified and highlighted in the data. These marked sections were grouped and analysed, leading to the creation of codes. The analysis of the identified codes was continued, and themes were formed by combining them (Braun and Clarke 2006). The initial coding and theme development were conducted by the first author. Subsequently, the other authors reviewed and commented on the analysis, and modifications were made based on this collaborative discussion to enhance the credibility of the findings.

Some studies focused on the same intervention; therefore, each intervention was described once in the TIDieR table, with all pertinent articles cited as references. Not all the aforementioned information was available in the articles; therefore, additional sources were used. For instance, some articles summarised results from previously detailed studies, or information was obtained from programme websites. A combination of narrative synthesis (Popay et al. 2006) and tables was used for data presentation.

## 4 Results/Findings

### 4.1 Details of the Selected Studies

The studies included in this review (Table 3) were conducted in 2020 (*n* = 3), 2021 (*n* = 7), 2022 (*n* = 4), 2023 (*n* = 6) and 2024 (*n* = 3). Geographically, studies were conducted in Australia (*n* = 5), the US (*n* = 4), the UK (*n* = 4), Canada (*n* = 2), Sweden (*n* = 2), Germany (*n* = 2), Denmark (*n* = 1), Spain (*n* = 1), Finland (*n* = 1) and the Netherlands (*n* = 1). The research designs included randomised controlled trials (RCTs) (*n* = 15, including one pilot), cohort studies (*n* = 2), a combination of RCT and cohort study (*n* = 1), cross‐sectional studies (*n* = 2, including one pilot), a cross‐sectional and register study (*n* = 1, pilot), a quasi‐experimental study (*n* = 1) and a single‐case experimental study (*n* = 1).

The reviewed studies examined various programmes and interventions, including Nurse‐Family Partnership (NFP; *n* = 2), Family Nurse Partnership (FNP; *n* = 3), Pro‐Kind (*n* = 2), VoorZorg (*n* = 1), right@home (*n* = 5), Family Connects (FC; *n* = 2), Newborn Behavioural Observations (NBO; *n* = 1), Video‐feedback Intervention (VIPP‐SD; *n* = 1), PUEDES Infancy Programme (*n* = 1), Minding the Baby (MTB; *n* = 1), Families First (*n* = 1) and INSIGHT (*n* = 1). Additionally, two studies investigated extended postnatal home visiting programmes without a specific programme name. The majority were home visit interventions (*n* = 19), of which 15 were targeted and four were universal. Nurse‐led parenting programmes were less common (*n* = 4), with one being targeted and three universal, two of which also included home visits. Notably, NFP, FNP, Pro‐Kind and VoorZorg originated from the same foundational programme but were adapted to fit specific cultural contexts. For example, in the UK, FNP was tailored to British nursing practices (Cavallaro et al. 2024; Robling et al. 2022; Waters et al. 2022); in Germany, Pro‐Kind was embedded in the collaboration between health and social services (Conti et al. 2021; Kliem and Sandner 2021); and in the Netherlands, VoorZorg addressed the needs of immigrant families, including language barriers and culturally sensitive engagement (van der Stouwe et al. 2023).

### 4.2 Main Findings of the Selected Studies

Overall, the interventions demonstrated positive outcomes for both parents and children. Home visiting interventions frequently improved parenting practices, including more positive parent–child interactions, greater maternal sensitivity and routine consistency (Conti et al. 2021; Baziyants et al. 2023; Goldfeld et al. 2022; Tauriello et al. 2023). The benefits for children included enhanced language development, executive functioning and school readiness (Robling et al. 2022; Catherine et al. 2024; Nguyen et al. 2024; Price et al. 2023) and fewer externalising and conduct problems (Waters et al. 2022; van der Stouwe et al. 2023; Tauriello et al. 2023; O'Farrelly et al. 2021). Interventions targeting parental mental health improved depression, anxiety, stress and overall well‐being (Kliem and Sandner 2021; Goldfeld et al. 2022, 2021), contributing to better parenting and fewer behavioural problems in children. Other effects included increased screening for postnatal depression, child vaccination (Chin et al. 2023) and reduced child protection involvement and emergency care use (Goodman et al. 2021).

Parenting interventions offer added value through peer support and increased parental self‐understanding (Sourander et al. 2021). Some also improved maternal knowledge of infant communication (Kristensen et al. 2020), self‐efficacy (Ruiz‐Zaldibar et al. 2021) and secure attachment behaviours (Slade et al. 2020), though some effects attenuated over time (Ruiz‐Zaldibar et al. 2021).

Tailored interventions for adolescent mothers, substance users and families needing interpreters have also demonstrated benefits, including reduced prenatal substance use (Catherine et al. 2020), improved child health literacy (Mekhail et al. 2023) and enhanced maternal satisfaction (Kanda et al. 2022). The evidence supports the effectiveness of early, structured and multicomponent interventions in promoting family well‐being across diverse delivery formats, particularly when initiated during pregnancy and continued into early childhood (Cavallaro et al. 2024; Waters et al. 2022; Catherine et al. 2024, 2020; Price et al. 2023).

Overall, the effect sizes varied considerably. Strong effects were observed, particularly in programmes targeting maternal substance use (Catherine et al. 2020), child language development (Catherine et al. 2024; Nguyen et al. 2024) and reductions in child maltreatment and emergency care use (Goodman et al. 2021). Moderate effects were noted in mental health outcomes such as depression, anxiety and stress (Goldfeld et al. 2021), whereas some interventions, such as FNP, showed only modest or mixed effects on child development (Cavallaro et al. 2024) and maternal outcomes, including language production (Waters et al. 2022). In a few cases, such as parental self‐efficacy (Ruiz‐Zaldibar et al. 2021) or maternal knowledge of infant communication (Kristensen et al. 2020), the initial gains diminished over time. Outcome details are presented in Table 3.

### 4.3 Details of the Interventions

#### 4.3.1 The Rationale and Theoretical Framework for the Interventions (Why)

Home visit and parenting interventions shared recurring goals. The most frequently mentioned objective was to improve children's health and development (Cavallaro et al. 2024; Robling et al. 2022; Waters et al. 2022; Conti et al. 2021; Kliem and Sandner 2021; Tauriello et al. 2023; Catherine et al. 2024, 2020; Slade et al. 2020). Another prominent theme was the enhancement of parenting skills and practices (Conti et al. 2021; Kliem and Sandner 2021; Baziyants et al. 2023; Catherine et al. 2024, 2020; O'Farrelly et al. 2021; Goodman et al. 2021; Kristensen et al. 2020; Ruiz‐Zaldibar et al. 2021). In addition, many interventions aimed at improving pregnancy and birth outcomes (Cavallaro et al. 2024; Robling et al. 2022; Waters et al. 2022; Catherine et al. 2024, 2020; Chin et al. 2023), support families by connecting them with resources (Baziyants et al. 2023; Goldfeld et al. 2022, 2021; Nguyen et al. 2024; Price et al. 2023; Goodman et al. 2021; Kanda et al. 2022) and prevent child maltreatment (van der Stouwe et al. 2023; Baziyants et al. 2023; Goodman et al. 2021). Economic self‐sufficiency (Cavallaro et al. 2024; Robling et al. 2022; Waters et al. 2022; Catherine et al. 2024, 2020), mental health and well‐being (Baziyants et al. 2023; Goodman et al. 2021; Sourander et al. 2021; Slade et al. 2020) and the promotion of equality and participation (Chin et al. 2023; Mekhail et al. 2023) were also significant goals across the various programmes.

Human ecology theory (Bronfenbrenner 1979) was the most frequently used theoretical framework (Cavallaro et al. 2024; Robling et al. 2022; Waters et al. 2022; Conti et al. 2021; Kliem and Sandner 2021; van der Stouwe et al. 2023; Baziyants et al. 2023; Goldfeld et al. 2022, 2021; Tauriello et al. 2023; Catherine et al. 2024, 2020; Nguyen et al. 2024; Price et al. 2023; Goodman et al. 2021; Kanda et al. 2022). Attachment (Bowlby 1969) (Cavallaro et al. 2024; Robling et al. 2022; Waters et al. 2022; Conti et al. 2021; Kliem and Sandner 2021; van der Stouwe et al. 2023; Catherine et al. 2024, 2020; O'Farrelly et al. 2021; Sourander et al. 2021; Kristensen et al. 2020; Slade et al. 2020) and self‐efficacy theory (Bandura 1977) (Cavallaro et al. 2024; Robling et al. 2022; Waters et al. 2022; Conti et al. 2021; Kliem and Sandner 2021; van der Stouwe et al. 2023; Catherine et al. 2024; Ruiz‐Zaldibar et al. 2021) were also widely applied. The frameworks enabled culturally sensitive, holistic interventions by integrating the environmental, relational and individual determinants of family well‐being. Additionally, personal construct theory (Kelly 1955), theories of helper characteristics and helping processes (Egan 1982; Rogers 1959), the synactive theory of development (Als 1982), the social learning theory (Bandura 1961) and the mentalisation framework (Fonagy and Bateman 1991) have been used. The two extended postnatal home visiting interventions lacked theoretical grounding.

#### 4.3.2 The Procedures and Materials Used (What)

Nurse‐ and midwife‐led interventions varied in recruitment, target groups and intervention methodologies. Some interventions adopted a broad recruitment approach with no strict eligibility criteria to enhance accessibility (Baziyants et al. 2023; Goodman et al. 2021; Sourander et al. 2021; Kristensen et al. 2020). Others targeted first‐time parents, recognising the unique challenges associated with the transition to parenthood (Cavallaro et al. 2024; Robling et al. 2022; Waters et al. 2022; van der Stouwe et al. 2023; Tauriello et al. 2023; Catherine et al. 2024, 2020; Slade et al. 2020). Several interventions focused on socially and economically disadvantaged families to reduce disparities in child development and parental well‐being (Conti et al. 2021; Kliem and Sandner 2021; van der Stouwe et al. 2023; Goldfeld et al. 2022, 2021; Nguyen et al. 2024; Price et al. 2023; Ruiz‐Zaldibar et al. 2021; Kanda et al. 2022). Additionally, some interventions specifically addressed families facing additional risk factors such as low educational levels, mental health difficulties and substance abuse (van der Stouwe et al. 2023; Goldfeld et al. 2022, 2021; Nguyen et al. 2024; Price et al. 2023; O'Farrelly et al. 2021; Slade et al. 2020; Kanda et al. 2022).

The materials and methods reflected diverse theoretical and practical approaches. Some interventions adhered to standardised protocols, ensuring consistency in their delivery (Conti et al. 2021; Kliem and Sandner 2021; Tauriello et al. 2023; Catherine et al. 2024, 2020). Similarly, structured guidelines provided clear frameworks for assessing and addressing the needs of parents and children. These guidelines ensured that interventions were evidence‐based with specific recommendations for each visit to effectively support parents (Baziyants et al. 2023; Goldfeld et al. 2022, 2021; Catherine et al. 2024, 2020; Nguyen et al. 2024; Price et al. 2023; O'Farrelly et al. 2021; Chin et al. 2023; Goodman et al. 2021; Kanda et al. 2022).

#### 4.3.3 The Delivery Methods (How)

All programmes primarily used face‐to‐face home visits (Cavallaro et al. 2024; Robling et al. 2022; Waters et al. 2022; Conti et al. 2021; Kliem and Sandner 2021; van der Stouwe et al. 2023; Baziyants et al. 2023; Goldfeld et al. 2022, 2021; Tauriello et al. 2023; Catherine et al. 2024, 2020; Nguyen et al. 2024; Price et al. 2023; O'Farrelly et al. 2021; Chin et al. 2023; Kristensen et al. 2020; Slade et al. 2020; Mekhail et al. 2023; Kanda et al. 2022). One programme added a final phone contact (Goodman et al. 2021), and two included group meetings, although the locations were not specified (Sourander et al. 2021; Ruiz‐Zaldibar et al. 2021). Group‐based interventions emphasised peer support and collective learning, offering parents opportunities to share experiences, normalise challenges and build social networks in a supportive environment (Kristensen et al. 2020; Slade et al. 2020).

A common foundation across many interventions was the use of strength‐based approaches, emphasising collaboration and built on existing family capacities (Caiels et al. 2021). Some programmes have systematically assessed family strengths and the need to provide support (Slade et al. 2020; Kanda et al. 2022), promoting engagement and resilience, particularly among high‐risk populations. This orientation provides a framework for implementing more specific change strategies.

Within this framework, motivational interviewing was used in several interventions to support individualised goal setting and behavioural change (van der Stouwe et al. 2023; Goldfeld et al. 2022, 2021; Nguyen et al. 2024; Price et al. 2023; Slade et al. 2020; Kanda et al. 2022). Some combined motivational interviewing with video feedback, which provided parents with real‐time, interactive guidance on their caregiving practices and supported the development of positive parenting behaviour change (Goldfeld et al. 2022, 2021; Nguyen et al. 2024; Price et al. 2023; Kanda et al. 2022).

Empowerment and confidence building strategies were central to many interventions. These approaches aim to strengthen parental self‐efficacy and participation (Chin et al. 2023), enhance parenting confidence through experiential learning (O'Farrelly et al. 2021), and support intuitive parenting by tailoring the content to each family's specific needs (Kristensen et al. 2020). Behavioural strategies and feedback were widely used to promote responsive parenting and behavioural regulation. Some programmes taught parents to recognise and respond to their child's cues (Goldfeld et al. 2021), while others used video and observational feedback to enhance parent–child interaction and deepen parents' understanding of their child's developmental needs (Goldfeld et al. 2022; Nguyen et al. 2024; Price et al. 2023; Kanda et al. 2022).

#### 4.3.4 The Timing and Dosage of the Interventions (When and How Much)

Many interventions begin during pregnancy or within the first few weeks of a child's life (Cavallaro et al. 2024; Robling et al. 2022; Waters et al. 2022; Conti et al. 2021; Kliem and Sandner 2021; Catherine et al. 2024, 2020; Slade et al. 2020). The number of visits ranges from a few to over 50, with regular home visits occurring weekly, biweekly or monthly (Conti et al. 2021; Kliem and Sandner 2021; van der Stouwe et al. 2023). Several interventions extended support beyond the first year of life. Interventions often continued until the child was 2 years old (Cavallaro et al. 2024; Robling et al. 2022; Waters et al. 2022; Catherine et al. 2024, 2020; Slade et al. 2020). Most included studies focused on children under the age of 5. Interventions targeting older children and adolescents were not represented in the included studies. Combining health, developmental and social support addressed various aspects of family needs (Goldfeld et al. 2022, 2021; Nguyen et al. 2024; Price et al. 2023; Kanda et al. 2022).

#### 4.3.5 The Locations (Where)

Most interventions were home‐based, emphasising the importance of providing support in the familiar and comfortable environment of the child's home (Conti et al. 2021; Kliem and Sandner 2021; van der Stouwe et al. 2023; Baziyants et al. 2023; Goldfeld et al. 2022, 2021; Tauriello et al. 2023; Catherine et al. 2024, 2020; Nguyen et al. 2024; Price et al. 2023; O'Farrelly et al. 2021; Chin et al. 2023; Goodman et al. 2021; Kristensen et al. 2020; Slade et al. 2020; Mekhail et al. 2023; Kanda et al. 2022). One intervention was primarily home based, with occasional visits to other locations (Cavallaro et al. 2024; Robling et al. 2022; Waters et al. 2022). Some interventions used group settings to foster peer support and collective learning experiences (Sourander et al. 2021). One intervention did not specify the interventions' locations (Ruiz‐Zaldibar et al. 2021).

#### 4.3.6 The Individuals Who Provided the Interventions (Who)

Most interventions were delivered by registered nurses with public or child health training and a bachelor's degree (Goldfeld et al. 2022, 2021; Tauriello et al. 2023; Catherine et al. 2024, 2020; Nguyen et al. 2024; Price et al. 2023; Kanda et al. 2022); some were implemented through interdisciplinary collaboration, often involving social workers (Conti et al. 2021; Kliem and Sandner 2021; Chin et al. 2023; Slade et al. 2020; Mekhail et al. 2023). Additional programme‐specific training was often required. Training included reading detailed manuals, observing experienced practitioners, participating in supervised practice sessions and passing assessments to ensure adherence to intervention models (Baziyants et al. 2023; O'Farrelly et al. 2021; Goodman et al. 2021; Slade et al. 2020). Certification was commonly required before nurses could begin to deliver their services (van der Stouwe et al. 2023; Baziyants et al. 2023; Goodman et al. 2021; Kristensen et al. 2020). Interventions frequently incorporated ongoing supervision, regular fidelity checks and opportunities for continuous professional development to maintain fidelity (Baziyants et al. 2023; Goldfeld et al. 2022, 2021; Nguyen et al. 2024; Price et al. 2023; Goodman et al. 2021; Slade et al. 2020; Kanda et al. 2022). Some nurses had postgraduate training or specialised modules in parenting, child development or family support (Cavallaro et al. 2024; Robling et al. 2022; Waters et al. 2022; Goldfeld et al. 2022, 2021; Nguyen et al. 2024; Price et al. 2023; Kanda et al. 2022).

#### 4.3.7 Customisation (Tailoring)

Many interventions emphasise personalised support tailored to interventions to meet each family's unique needs (Cavallaro et al. 2024; Robling et al. 2022; Waters et al. 2022; Conti et al. 2021; Kliem and Sandner 2021; Baziyants et al. 2023; Goldfeld et al. 2022, 2021; Catherine et al. 2024, 2020; Nguyen et al. 2024; Price et al. 2023; Chin et al. 2023; Goodman et al. 2021; Kristensen et al. 2020; Slade et al. 2020; Kanda et al. 2022). Interventions adapted both the structure and content of home visits to each family's unique needs and circumstances. This process typically began with an initial assessment and continued throughout the intervention, thereby allowing professionals to respond flexibly to family feedback and evolving situations. Strategies included agenda‐matching, adjusting visit frequency and referring families to appropriate community services. Some programmes used multidisciplinary teams for coordinated support. Cultural adaptations were also implemented to ensure that the interventions aligned with local healthcare systems, professional roles and societal norms. These included modifying staffing models, altering session frequency and adapting materials or delivery formats to better fit cultural and institutional contexts (van der Stouwe et al. 2023; O'Farrelly et al. 2021). Group sessions were also used in interventions to foster peer support and collective learning (Sourander et al. 2021). A few interventions did not specify customisation in their descriptions (Tauriello et al. 2023; Ruiz‐Zaldibar et al. 2021; Mekhail et al. 2023).

#### 4.3.8 Content of the Interventions

The interventions presented above provided a wide range of services and strategies, primarily focused on improving the health and well‐being of mothers, infants and families. Thematic analysis of the key content components revealed several recurring themes organised around five core themes: (1) parenting education, (2) maternal and infant health, (3) mental health and psychosocial support, (4) community connections and resources and (5) cultural and contextual sensitivity (Table 5).

**TABLE 5:** Key components of the program content.

Theme	Code
1. Parenting education	Infant care and development
	Child development
	Parental role and responsibilities
2. Maternal and infant health	Health monitoring and guidance
	Lactation and feeding
	Mental health support
3. Mental health and psychosocial support	Psychosocial support and family dynamics
	Maternal reflective functioning
	Support systems
4. Community connections and resources	Referrals and support networks
	Social and health service access
5. Cultural and contextual sensitivity	Family diversity and individualised approaches
	Contextual factors in parenting

##### 4.3.8.1 Parenting Education

Parental education regarding infant care, including feeding practices, sleep routines and infant health, was crucial. Programmes aimed to strengthen parental knowledge by providing information on sleep, feeding and soothing, and enhance parenting skills through guidance on sensitive caregiving behaviours that support attachment and help foster secure emotional bonds with children (Baziyants et al. 2023; Tauriello et al. 2023; O'Farrelly et al. 2021; Chin et al. 2023; Goodman et al. 2021; Mekhail et al. 2023). In addition to practical caregiving, several interventions focused on developing parental understanding of child developmental stages, discussing developmental milestones, encouraging positive parent–child interactions and managing behaviours such as crying and fussiness. Improving parent–infant interactions by enhancing awareness of infant competencies and neurobehavioural regulation was also emphasised (Chin et al. 2023; Kristensen et al. 2020; Ruiz‐Zaldibar et al. 2021; Slade et al. 2020). Furthermore, many programmes addressed parental attitudes and roles, supporting the transition to parenthood by exploring maternal and paternal responsibilities, child safety and the importance of nurturing and responsive interactions (Conti et al. 2021; Kliem and Sandner 2021; Catherine et al. 2024, 2020; Chin et al. 2023).

##### 4.3.8.2 Maternal and Infant Health

The interventions offered health screenings, check‐ups and advice on maternal and infant health, covering critical aspects such as breastfeeding, nutrition, maternal mental health and healthcare utilisation. Nurses typically provide guidance on infant weight, vital signs and other health factors (Baziyants et al. 2023; Catherine et al. 2024, 2020; Chin et al. 2023; Goodman et al. 2021; Sourander et al. 2021; Mekhail et al. 2023). Additionally, many interventions provided specific support for lactation, teaching techniques for breastfeeding, managing milk supply and addressing challenges such as bottle feeding and pumping. This ensured that the mothers had access to practical support for infant feeding and nutrition (Baziyants et al. 2023; Tauriello et al. 2023; Chin et al. 2023; Goodman et al. 2021) Furthermore, maternal mental health support was a common feature of multiple interventions. This included screening for postpartum depression, offering strategies to cope with stress, and conducting reflective discussions to improve maternal well‐being. Mental health support addressed issues such as stress management, coping with parental anger and managing PTSD symptoms (van der Stouwe et al. 2023; Chin et al. 2023; Sourander et al. 2021; Slade et al. 2020; Mekhail et al. 2023).

##### 4.3.8.3 Mental Health and Psychosocial Support

The interventions addressed the emotional challenges faced by parents, focusing on stress, anger, trauma and family relationships. They provided guided discussions and therapy referrals for managing negative emotions and encouraged reflection on family interactions (van der Stouwe et al. 2023; Chin et al. 2023; Sourander et al. 2021; Slade et al. 2020; Mekhail et al. 2023). Additionally, a strong emphasis was placed on reflective parenting. Parents were encouraged to reflect on their emotions, behaviours and their child's needs, which helped improve their own well‐being and relationship with their child (Chin et al. 2023; Sourander et al. 2021; Slade et al. 2020). Furthermore, building a supportive network for the family was emphasised, including involvement with partners, extended families and friends. This helped parents feel supported by both their parenting roles and daily challenges (Conti et al. 2021; Kliem and Sandner 2021; Chin et al. 2023; Sourander et al. 2021).

##### 4.3.8.4 Community Connections and Resources

Families were connected to healthcare services, food assistance programmes and mental health services through various interventions. These efforts were aligned with community‐based networks to provide comprehensive support (Baziyants et al. 2023; Chin et al. 2023; Goodman et al. 2021). Additionally, many initiatives helped families navigate social services and guided parents in utilising available health and community services. These referrals not only addressed the immediate needs of families but also aimed for sustainable, long‐term support in various areas of life (Conti et al. 2021; Kliem and Sandner 2021; Goldfeld et al. 2022, 2021; Nguyen et al. 2024; Price et al. 2023; Mekhail et al. 2023; Kanda et al. 2022).

##### 4.3.8.5 Cultural and Contextual Sensitivity

Recognising individual family dynamics, cultural differences and diverse parenting styles was emphasised. Parents were encouraged to reflect on their children's unique circumstances and specific needs (Chin et al. 2023; Sourander et al. 2021; Slade et al. 2020). In addition, broader social contexts, including economic factors, social support and access to resources, were addressed. Efforts aimed to reduce barriers to positive parenting by tackling external factors, such as stress and poverty (Conti et al. 2021; Kliem and Sandner 2021; van der Stouwe et al. 2023; Mekhail et al. 2023).

## 5 Discussion

Based on the analysis of nurse‐ or midwife‐led home visit and parenting interventions, the core components associated with positive outcomes for children and parents encompassed both practical implementation elements and thematic content components. Given the focus of this review on nurse‐led interventions, all the included programmes relied on qualified and trained nurses. A key strength of such interventions is their structured approach to training, certification and fidelity monitoring. These findings highlight that nurses receive rigorous preparation, including formal education, protocol‐specific certification, supervised practice sessions and continuous professional development. These processes are essential to ensure consistency, fidelity to intervention models and the delivery of high‐quality services.

Moreover, the incorporation of regular fidelity checks, interdisciplinary collaboration and structured supervision further strengthens the impact of these programmes by enhancing nurses' capacities to provide specialised health guidance, psychosocial support and tailored parenting strategies. Although this review focused on identifying individual components that could be more feasible to implement than comprehensive interventions, the importance of adequate education and professional competence among nurses cannot be overlooked. The content of the interventions was wide‐ranging, covering themes such as parenting support, maternal and infant health, mental health and psychosocial care, community engagement, structured delivery frameworks and cultural sensitivity. This underscores the need for nurses to possess diverse and specialised competencies to address families' unique needs effectively.

Several interventions identified in this scoping review used broad recruitment strategies without strict eligibility criteria, reflecting an inclusive approach to parenting support. Nevertheless, a significant proportion of the programmes adapted their implementation to the needs of specific subpopulations, indicating that a universal, one‐size‐fits‐all model may be insufficient. Targeted approaches were directed toward first‐time parents, socioeconomically disadvantaged families and individuals exposed to specific risk factors. This finding is especially pertinent in the context of Nordic welfare systems, such as those in Finland, where family social and health services are primarily organised as universal measures and offered to all individuals within the relevant target group. However, growing socioeconomic disparities in health outcomes suggest that existing services lack sufficient scale and intensity to adequately support at‐risk children and their families. This underscores the need for proportionate universalism, where interventions are universally available but delivered at a scale and intensity proportional to the level of need (Eklund Karlsson et al. 2022).

A key objective frequently highlighted in the reviewed interventions was the enhancement of children's health and development. This focus underscores the critical importance of early childhood as the foundation for long‐term health and well‐being (Britto et al. 2017). Effective interventions aim to provide comprehensive support for physical, cognitive and emotional development. By prioritising child health and development, these interventions seek to create a strong foundation for future success, reduce the risk of adverse outcomes and promote overall family well‐being (Jeong et al. 2021). The unifying factor among the objectives of the interventions was holistic support and empowerment of families. The human ecology (Bronfenbrenner 1979), attachment (Bowlby 1969) and self‐efficacy theories (Bandura 1977) were most frequently cited as guiding principles. These frameworks supported the design of holistic and culturally sensitive approaches that addressed the complex interplay of environmental, relational and individual factors that influence family well‐being. By promoting secure parent–child relationships, acknowledging contextual diversity and enhancing parental confidence and competence, interventions can effectively support diverse families and contribute to improved developmental and psychosocial outcomes (Salo et al. 2022; Younas and Gutman 2026).

The results related to practical implementation, specifically the procedures and materials used, delivery methods and timing and dosage of the interventions, revealed that interventions often emphasised an early start and regular visits. Many interventions began during pregnancy or within the first few weeks of the child's life and often continued until the child was 2 years old. The number of visits ranged from a few to more than 50. These results are consistent with previous findings (Casillas et al. 2016; Cibralic et al. 2025; Molloy et al. 2021; Beatson et al. 2021), reinforcing the importance of early initiation, frequent contact and sustained support in maximising the effectiveness of home visiting interventions.

Although the inclusion criteria allowed for studies involving children up to 16 years old, the interventions identified in this review predominantly targeted children in the early years. This reflects a broader trend in the literature where early intervention is prioritised because of its potential long‐term impact on child development and family well‐being (Britto et al. 2017). Most parenting programmes have been specifically designed for parents of children aged 3 to 10 (Jeong et al. 2021). However, in this review, the oldest children included in the interventions were 5 years old. This raises an important question regarding whether parenting interventions are adequately equipped to support the well‐being of older children and adolescents and highlights a gap in existing literature regarding interventions for older children and adolescents. It is important to note that many interventions were conducted in high‐income countries and often involved adaptations of the NFP model, such as FNP, VoorZorg and Pro Kind, which may have influenced the prominence of certain themes in the findings. This should be considered when interpreting the results, particularly in relation to their generalisability to low‐ and middle‐income contexts or to interventions based on entirely different models.

### 5.1 Implications for Practice and Future Research

This review highlighted the importance of equipping nurses and midwives with comprehensive training and ongoing support to deliver effective home visits and parenting interventions. Structured programmes that incorporate evidence‐based frameworks, cultural sensitivity and personalised approaches are essential to address the diverse needs of families. Health systems should prioritise the integration of these interventions into routine maternal and child health services to ensure equitable access and continuity of care. Emphasis should also be placed on proportionate universalism, where services are universally available but scaled according to need to reduce disparities in child and family outcomes.

Future studies should explore the long‐term sustainability and cost‐effectiveness of core components identified in this review, particularly in diverse healthcare settings. There is also a need to investigate how nurses and midwives can use digital and hybrid delivery models to complement traditional face‐to‐face interventions, particularly in underserved and remote areas. Additionally, more research is needed on interventions targeting older children and adolescents, as current programmes predominantly focus on children's early years. Comparative studies examining the effectiveness of universal versus targeted approaches and the role of interdisciplinary collaboration will further inform the development of scalable and adaptable intervention models.

### 5.2 Strengths and Limitations

This review adhered to the PRISMA guidelines, aligning with the current standards for scoping reviews. Two authors independently conducted the abstract and full‐text screenings, followed by consensus decisions, ensuring the reliability and validity of the final paper selection. An extensive literature search on home visiting and parenting interventions was carried out, encompassing various study designs. Previous reviews have not thematically examined the content of the interventions.

This review has several limitations that should be acknowledged. Data extraction was initially carried out by one reviewer, which may introduce bias. However, the process was subsequently reviewed and verified by the co‐authors to ensure accuracy and consistency. Qualitative studies were excluded to maintain a focus on robust, quantitative evidence and to support the synthesis of comparable, measurable outcomes necessary for identifying core components. While this approach enhances the clarity and consistency of the findings, it may limit the depth of understanding regarding contextual and experiential aspects of the interventions. Additionally, relevant studies may have been overlooked due to the exclusion of papers in languages other than English or Finnish. It should also be noted that several programmes are adaptations of the NFP (FNP, VoorZorg, Pro Kind), which may result in certain themes being more prominent than if entirely different programmes were considered. Digital interventions were excluded based on the eligibility criteria. This decision was made to maintain a clear focus on face‐to‐face or hybrid models involving direct interaction with nurses or midwives. Athough this approach enhances the specificity of the findings, it may limit the generalisability of the results to an increasingly digitalised healthcare landscape. Future reviews should explore how digital components complement or replace traditional delivery methods in parenting support.

## 6 Conclusion

This scoping review identified the core components of effective home visits and parenting interventions delivered by nurses and midwives. These findings underscore the importance of early, structured and personalised support that addresses families' multifaceted needs. Interventions that integrate parenting education, maternal and infant health, mental health support, community connections, structured frameworks and cultural sensitivity have been associated with improved outcomes for both children and parents. This review highlights the critical role of trained nurses and midwives in delivering these interventions and the value of tailoring services to meet the diverse needs of families. These insights provide a foundation for strengthening existing programmes and guiding the development of future interventions that are both effective and scalable. Importantly, the evidence reviewed focused primarily on the early years of life, and there is a gap in interventions for older children and adolescents. In the future, programme development and research should aim to cover a broader age range to ensure that support is continuous, developmentally appropriate and impactful throughout the different stages of childhood and adolescence.

## Funding

This work was supported by the Finnish Association of Nursing Research (HTTS ry.) under Grant 2025. Recipient: Outi Savolainen.

## Conflicts of Interest

The authors declare no conflicts of interest.

## Supporting information


**Table S1:** Characteristics of interventions.

## Data Availability

Data sharing not applicable to this article as no datasets were generated or analysed during the current study. Trial and protocol registration: Open Science Framework (https://osf.io/), DOI: https://doi.org/10.17605/OSF.IO/EN4PS.
