# Supplementary material for: Core Components of Effective Home Visiting Programmes and Parenting Interventions Delivered by Nurses and Midwives—A Scoping Review
Source: J Adv Nurs. 2026 Jan 21;82(9):8391–412. doi: 10.1111/jan.70494 (PMC13460992; doi:10.1111/jan.70494)
Supplement: Supplementary file 1 [file JAN-82-8391-s001.docx]

**Supplementary Table 1.** Characteristics of interventions.

| **Study authors** | | **Intervention** | **Why**  Rationale and theoretical framework | **What**  Procedures and materials used | **How**  Delivery methods | **When and how**  **much**  Timing and dosage | **Where**  Locations | **Who**  Individuals who provided the interventions | **Tailoring** |
| --- | --- | --- | --- | --- | --- | --- | --- | --- | --- |
| **Universal home visiting programmess/interventions** | | | | | | | | | |
| Baziyants et al. (2023), Goodman et al. (2021) | | Family Connects (FC) | To prevent child maltreatment by supporting all families in a community through one to three visits to improve parent mental health and parenting behaviors.  To identify family-specific needs, deliver education and intervention, and connect families with community resources matched to their needs.  Human Ecology Theory (Bronfenbrenner, 1979) (1) | Participants are recruited ideally in a hospital shortly after birth. Programme staff attempted to engage and enroll all the families in the intervention.  No eligibility criteria apart from having given birth  Nurse screens the family to identify family strengths and family needs during the initial visit. Nurses address each need by providing brief interventions or connecting the family to various community agencies. Community alignment brings together the hundreds of agencies in a community that serve birthing families through an electronic directory and community advisory boards. An integrated data system links families and community services.  The FC programme adheres to a manualized protocol consisting of the following three core components: the direct intervention consists of engagement with families shortly after birth, assessment and intervention with families through one to three home visits with a registered public health nurse, and a final telephone contact four weeks after the home visit(s). During in-home visits, nurses provide brief educational interventions for all families (e.g., feeding practices) and, using a high-inference approach combining parent self-report, direct observation, and nurse clinical judgement, systematically assess family need across 12 empirically derived factors associated with child health and well-being (health care: maternal health, infant health, and health care plans; parenting and childcare: childcare plans, parent–child relationship, and management of infant crying; family material resources and safety: material supports, family and community safety, and birthing parent history of parenting difficulties; and parent well-being: mental health, substance abuse, and social-emotional support). | Face-to-face home visits to families and final telephone contact. | One to three home-visits over the first 12 weeks of life starting at child age of three weeks. | Child’s home | Registered public health nurse  Quarterly fidelity checks with all nurses to ensure proper adherence to the FC model  Programme requires training of each staff member, certification of nurses in the protocol, ongoing supervision, and documentation that implementation  adheres to the programme model. After qualified nurses are hired, their training begins with reading  detailed manuals. Then they observe expert nurses during home visits and conduct practice home visits that are observed by a supervisor. They are tested on how well hey adhere to the way questions are posed to parents and how reliably they score risk in each of the 12 domains. Finally, they receive certification that they are ready  to implement the programme. Nurse supervisors conduct quarterly fidelity checks with all home visiting nurses at their site (1). | Nurses address each need by providing brief interventions or connecting the family to various community agencies. |
| Kristensen et al. (2020) | | Newborn Behavioural Observations (NBO) | To enhance the parents’ understanding of the newborn’s cues and thereby respond sensitively to their newborn’s expressions and cues.  Attachment Theory (Bowlby 1969)  Synactive Theory of Development (Als 1982) (2) | Recruiting: Health visitors (RN) at the first home visit after birth.  No exclusion criteria except mothers who were undergoing treatment elsewhere and not living at home or who were unable to manage their own legal affairs and therefore not visited by a health visitor.  Families received NBO intervention practiced by the health visitors during the home visit when the infant was three weeks old and in any subsequent home visit until the age of three months.  The intervention focuses on observation of the infant combined with active involvement of the parents in a shared dialogue based on 18 neuro-behavioural items including both observation and elicited manoeuvers to identify newborn behaviours and interpreting these in the context of parent-infant interaction. The items include infant habituation to stimuli, infant motor development, observation of the infant’s state regulation (consciousness from deep sleeping to crying) and response to face, voice, stress and activity using the NBO system. | Face-to-face home-visits to families  Observation-based parent feedback session | Families received NBO intervention practiced by the health visitors during the home visit when the infant was three weeks old and in any subsequent home visit until the age of three months. The  NBO observation takes around 12 to 25 min. | Child’s home | Registered nurse.  Two Danish NBO trainers certified by the Brazelton Institute, UK, delivered the NBO education and supervision to all health visitors employed in the intervention districts.Supervision was given to health visitors in the intervention group during the implementation period for a total of 2 days to ensure that the delivery of NBO did not deviate from the standardised NBO system. | Administration of the NBO is flexible; if the infant  sleeps at the beginning of the session, then the NBO be-  gins with the discussion of habituation issues. If, however,  the infant is crying, then the session begins with observa-  tion of infants state regulation and soothability. |
| **Targeted home visiting programmes/interventions** | | | | | | | | | |
| Catherine et al. (2020), Catherine et al. (2024) | | Nurse-Family Partnership (NFP) | To improve child and maternal well-being. The aims are to develop a close, trusting partnership to improve: pregnancy outcomes through positive maternal health behaviours; child health and developmental outcomes through sensitive and competent parenting; and maternal economic self-sufficiency through pregnancy spacing and encouragement of education and employment opportunities.  Self-Efficacy Theory (Bandura, 1977)  Human Ecology Theory (Bronfenbrenner, 1979)  Attachment Theory (Bowlby 1969) | Nurses contacted those allocated to the intervention arm to schedule the first home visit before 29 weeks gestation.  Girls and young women who were preparing to parent for the first time, were experiencing socioeconomic disadvantage and were aged 24 years or younger.  Visits followed NFP guidelines: NFP Visit-to-Visit guideline materials). Nurses assist participants to identify and meet health and social goals (incl. substance use) (3). | Face-to-face home-visits to families | From early pregnancy until children reach age two years. Fourteen visits prenatally, 50 postpartum. | Child’s home | Public health nurses – registered nurses with a bachelor’s degree in nursing at a minimum.  Nurses completed NFP education (appr. 149 hours and ongoing learning). | Although NFP nurses have a structured set of guidelines to guide the content of each visit, they adapt them as needed to address the individual and unique needs/circumstances of each family (agenda-matching) (3). |
| Cavallaro et al. (2024), Robling et al. (2022), Waters et al. (2022) | | Family Nurse Partnership (FNP)  (Nurse Family Partnership’ NFP is the original programme name in the USA) | To improve birth outcomes, child health and development and promote economic self-sufficiency among young mothers.  FNP aims to improve pregnancy outcomes by enabling young women to improve their antenatal health and the health of their unborn baby  - children’s subsequent health and development by supporting parents to provide consistent, competent care for their children  - women’s life course by planning subsequent pregnancies, and supporting access to education, training and employment (4).  Attachment Theory (Bowlby 1969)  Human Ecology Theory (Bronfenbrenner 1979) Self-Efficacy Theory (Bandura 1982) | Family Nurse Partnership teams recruit first-time mothers. The aim is to enrol clients on the programme as early as possible in pregnancy (ideally before 16 weeks’ and ordinarily no later than 28 weeks and 6 days’ gestation) to enable key work to be done to support healthy pregnancy and to prepare for the transition to parenthood (4).  Mothers aged 13–19 at last menstrual period who had their first live birth between 1 April 2010 and 31 March 2019 and whose first antenatal booking appointment as recorded in HES (or estimated date of 28 weeks gestation, if missing) occurred on a date when the FNP was active in their local authority of residence.  A variety of evidence-based tools, methods and materials are used (4). | Face-to-face home-visits to families | Up to 64 home visits from early pregnancy until the child’s second birthday. The mean number of visits received by mothers across the programme was 36 (median 38). | Child’s home  FNP is a home-based visiting programme but family nurses will occasionally meet clients in another appropriate location - for example, a GP surgery (4). | Dedicated Family Nurse  a specially trained family nurse (4). | Families will be supported to access wider formal and informal support to meet their individual needs (4). |
| Chin et al. (2023) | | Extended postnatal home visiting programme 1 | To strengthen the self-confidence of parents and to promote their trust in antenatal care and children and child healthcare as well as social services. Furthermore, the intervention aimed to promote equality and participation among the parents and to identify families with extra professional support needs at an early stage.  Does not mention background theories, but refers to attachment theory, ecological systems theory, empowerment theory, and salutogenesis. | Midwives at maternal healthcare centre invited parents to participate in the intervention during a prenatal assessment between the 29th and 32nd week of pregnancy.  The parents lived in one of the two included regions and that they were expecting their first child to be born in Sweden (i.e. the parents could have had another child previously in another country). | Face-to-face home-visits to families | Four extra home visits at 2, 4, 12, and 15 months of age. The first home visit during week 34 of the pregnancy. home visits were then offered in week 1–2 and when the child was 2, 4, 8 and 15 months old. | Child’s home | Parent advisor from the social services and a midwife. | The intervention was designed to be personalized and tailored to the specific needs of families. The personalization process began with an initial prenatal assessment by a midwife between the 29th and 32nd week of pregnancy, which helped identify the family's needs. The content of the visits was flexible, allowing professionals to adjust their focus based on the family's evolving needs and feedback. |
| Conti et al. (2021), Kliem & Sandner (2021) | | Pro-Kind  (the German adaptation of the Nurse-Family Partnership (NFP)) | To promote child development by strengthening the intuitive parenting skills of mothers.  Attachment Theory (Bowlby 1969)  Human Ecology Theory (Bronfenbrenner 1979) Self-Efficacy Theory (Bandura 1982) | The participants were recruited through various multipliers – especially through gynaecologists, midwives, youth welfare offices, psychosocial counselling centres,  and employment agencies (5).  Financially and socially disadvantaged with at least a basic understanding of the German language.  Teaching materials and visit-by-visit guidelines, adapted from NFP, structure the aim and content of each home visit.  As part of the study, visit content was recorded by the nurses at the completion of each visit. The nurses completed an online checklist designed explicitly for use in programme quality monitoring. It included the unique client identifier, visit date, and the activities undertaken. The electronic checklist was located on the nurse’s mobile device (tablet) with a simple touch entry. There were 48 content items in the antenatal checklist and 56 content items in the postnatal checklist. | Face-to-face home-visits to families | Intervention starts between the 12th and 28th week of pregnancy and continues until the child’s second birthday.  The frequency of the home visits varies between weekly, biweekly, and monthly according to the NFP model, for an overall maximum of 52 home visits with an average duration of 90 min each. | Child’s home | In contrast to NFP, where nurses conducted the home visits, mainly family midwives and social pedagogues conducted the home visits alone or in a team.  Both family midwives and nurses hold a  three-year apprenticeship degree consisting of 1,600 h of theory and 3,000 h of practical training, while social pedagogues hold a  university BA degree. | The Pro Kind intervention was personalized to meet the unique needs of each mother-child dyad. This personalization involved tailoring the frequency and content of home visits and the strategies used. The personalization process began during the initial home visits with baseline assessments and continued throughout the intervention. Regular assessments by trained professionals facilitated modifications to the intervention, including changes in visit frequency, new activities to boost positive interactions, and additional resources for identified challenges. |
| Goldfeld et al. (2021), Goldfeld et al. (2022), Kanda et al. (2022), Nguyen et al. (2024), Price et al. (2023) | | right@home | To support mothers experiencing adversity.  Human Ecology Theory (Bronfenbrenner, 1979), Personal Construct Theory (Kelly 1955), theories  of helper characteristics and helping processes (Egan 1982, Rogers 1959) (6) | Women were recruited from antenatal clinics.  Women had due dates before October 1, 2014, were 37 weeks’ gestation, had sufficient English to complete face-to-face interviews, lived within travel boundaries specified by participating areas, and had 2 of 10 risk factors identified at screening (young pregnancy; not living with another adult; no support in pregnancy; poor health; a long-term illness, health problem, or disability that limits daily activities; currently smokes; stress, anxiety, or difficulty coping; low education; no person in the household currently earning an income; and never having had a job before).  Programme was structured around the core Miller Early Childhood Sustained Home-visiting framework and training and bolstered by five evidence-based strategies for content (sleep, safety, nutrition, regulation, and bonding and relationship) and two for the delivery process (video feedback and motivational interviewing strategies). Maternal mental health and well-being support (e.g., nurse-led discussion of maternal mood, coping, and identification of additional support needs) were offered as components within the programme). | Face-to-face home-visits to families  Motivational interviewand individualized intervention plan | Mothers in the intervention group were offered 25 nurse home visits (mean: 23.2 home visits received) 60–90 minutes, commencing antenatally and delivered mostly by the same nurse trained in the right@ home NHV model of care. Most intervention group women (75.6%) also received 1 or more than one home visits by a social care practitioner (mean: 1.7  visits),28 who provided brief counselling interventions and/or case management as needed. | Child’s home | Nurse. Some visits by social care practitioner.  The right@home nurses must be qualiﬁed CFH nurses, that is, Baccalaureate-registered nurses (or equivalent) with postgraduate qualiﬁcations in CFH. They must also have completed Family Partnership Model Training. Programme nurses undertook 4 h of online training,12 h of face-to-face training in the core MECSH programme, and an additional 7 h training in theright@home focus modules (6). | The personalized approach ensured that the support was tailored to each mother's unique circumstances. |
| Slade et al. (2020) | | Minding the Baby (MTB) | To improve health, developmental, attachment, mental health, and life course outcomes in vulnerable families.  Attachment Theory (Bowlby 1969) | Primiparous women attending prenatal care sessions at the community health centres were approached to assess their interest in participating in the study.  Inclusion criteria for mothers included the following: able to speak and understand English, between 14 and 25 years of age, having a first child, no active heroin or cocaine use (prescreened by the CHC as criteria for entry into prenatal care), no DSM-IV psychotic disorder; and (f) no major or terminal chronic condition in the mother (AIDS, cancer, etc. [prescreened by the CHCs])  Relationship-based programme. Both the nurse and the social worker provide developmental guidance, scaffold parenting, and support the developing mother–infant attachment relationship. They also attend to families’ physical safety and basic necessities, supplying diapers, emergency food assistance, and the like. The nurse focuses on health promotion (including reproductive care, nutritional guidance, fore-shadowing, and links to the medical home, and may screen for depression (typically postpartum depression) and suggest non-pharmacological interventions such as exercise and rest. | Face-to-face home-visits to families  Motivational interviewing techniques and individualized intervention plan | Weekly home visits in the late second or early third trimester of pregnancy. These continued until the child’s first birthday, when there was a celebratory ‘transition’ visit acknowledging the growth and progress of the first year and setting goals for visits in the second year. After this point, the home visits occurred every other week until the child was 24 months old. | Child’s home | Nurse and social worker team  Fidelity to the model was maintained through ongoing training and supervision. MTB practitioners first completed an introductory three-day training and became familiar with MTB’s comprehensive treatment manual. Once they began seeing families (a full-time caseload was 20–25 families), they met weekly for an hour of discipline-specific supervision (i.e. the nurse met with a nurse supervisor, the social worker with a social work supervisor), an hour of interdisciplinary supervision, with supervisors from both disciplines in attendance, and a 90-min team meeting. All supervision (typically provided by programme directors) incorporated case review, and clinical as well as reflective supervision. Clinicians also took part in quarterly professional training sessions led by programme directors. | The frequency of visits is adjusted according to each family’s need, with more frequent visits offered at times of crisis. |
| van der Stouwe et al. (2023) | | VoorZorg, (Intervention finds its origins in the Nurse- Family Partnership programme (NFP)) | To prevent child maltreatment in at-risk families  Self-Efficacy Theory (Bandura, 1977)  Human Ecology Theory (Bronfenbrenner, 1979)  Attachment Theory (Bowlby 1969) | Trained nurses invited mothers in their caseload to participate.  The mothers were eligible for VoorZorg when they are younger than 26 years, have a low educational level (i.e., dropped out of school or maximum pre-vocational secondary education), are pregnant with their first child, and have at least sufficient understanding of the Dutch language to be able to participate in the programme. Added intervention components: The mothers were eligible for participation if they had at least 36 weeks of VoorZorg intervention left (i.e., child aged 0–15 months) and sufficient understanding of the Dutch language to participate.  Intervention used the structured intervention components targeting perceived stress, parental anger, and posttraumatic stress disorder (PTSD) symptoms. The stress component consisted of a relaxation exercise audio clip for daily use, starting the first treatment home visit. Starting the second treatment phase home visit, anger triggers  and attributions and ways to calm down were discussed and normalized. The severity of PTSD symptoms was assessed in the fourth treatment phase home visit, and direct referral to (online) eye movement desensitization and reprocessing therapy (EMDR) was offered. | Face-to-face home-visits to families  Individualized intervention plan | Participants received regular (mostly biweekly) VoorZorg home visiting intervention for the full 36-week period. Started weekly during pregnancy and continued biweekly up from the seventh week after birth into the second life year of the child. | Child’s home | Trained VoorZorg Youth Health Care nurses. Qualified VoorZorgYouth Health Care nurses were trained in the additional intervention components. | Intervention finds its origins in the  NFP but has been culturally  adapted and further developed after implementation in The Netherlands in such a way that it is now regarded as a stand-alone intervention programme. |
| Mekhail et al. (2023) | | Extended postnatal home visiting programme 2 | To reduce child health inequalities  No mention of background theory | Participants are recruited through two child health care centres.  Participants’ children needed to be aged < 2 months at the time of recruitment. One or both parents from each family had a choice to participate. As language interpreters were available, speaking fluent Swedish/English was not required  Nurses’ focus within the programme is comprehensive health literacy issues, whereas parental advisors, who are also trained social workers, provide psychosocial support, especially regarding family relations and interactions. | Face-to-face home visits | An extended postnatal home visiting program/intervention during the child’s first 15 months to supplement the ordinary Swedish CHC programme. The programme includes six joint home visits by a CHC nurse and parental advisor from social services when the child is a newborn aged 2, 4, 8, 12 and 15 months. Each home visit lasts around one hr. the ordinary, universal Swedish CHC programme, which is free of charge to all families with children aged 0−5 years. The ordinary pro- gram includes one postnatal home visit to families with newborn babies. | Child’s home | Nurses and social workers. The programme follows written guidelines. nurses specializing in child health | N/A |
| **Universal parenting programmes/interventions** | | | | | | | | | |
| Ruiz-Zaldibar et al. (2021) | | PUEDES Infancy program | To develop a positive parenting, parenting styles and parenting skills regarding to children’s diet, exercise, and screen time, and two additional sessions about child development and family games.  Self-Efficacy Theory (Bandura 1982) | Participants were recruited from community associations  The inclusion criteria for participants were that they were parents (biological or not) of children between two and five years old, be older than 18, having signed the informed consent and able to communicate in Spanish.  The sessions were conducted in small groups of 8–10 participants. Each session involved three different phases given the theoretical grounding of the study based on Bandura’s theory of self-efficacy, positive parenting and parenting styles. The programme focused on building parental self-efficacy through experiential activities related to child development, lifestyles (diet, exercise, and screen view) and family games. | Group sessions, the place is not mentioned  Peer support activities | Six 2-h group sessions held once a week (120 min per session), 2two- to five-year-old children | The place is not mentioned | The sessions were led by a Registered Nurse trained in leading small groups and parenting. | N/A |
| Sourander et al. (2021) | | Families First (based on a format called Parents First) | To enhance parental mentalization capacity and sensitivity in parent–child interaction (dyadic approach). To promote communication between family members, to create  conditions for healthy, secure relationships within the family (triadic approach), to build up sufficient resilience to withstand future adversities (health promotion approach); to provide parents an opportunity to establish a strong social support network that will persist after the intervention is over (peer-support approach); to reach a large proportion of the population, while at the same time reduces expenditures and strain on special healthcare.  Mentalization (Fonagy & Bateman 1991),  Attachment Theory (Bowlby 1969) | Nurses working in the well- baby clinics invited first-time parents  No eligibility criteria, except being a first-time parent  Families First groups were mainly organized by well-baby clinics as a continuation of prenatal education classes. Two group leaders held Families First sessions every other week. In addition to the group sessions, the Parents First format included individual consultations as well as educational hand-outs on child development. Families First intervention would comprise 12 group sessions. When the Families First group sessions ended, the children were usually nine- or 10- months old. Two trained group leaders, forming a working couple, together lead each group. A maximum of four to six families can participate, making a total of 12 to 18 participants, plus the two group leaders. | Face-to-face group meetings  Peer support activities | Two group leaders held Families First sessions (two hours) every other week. In addition to the group sessions, the Parents First format included individual consultations as well as educational hand-outs on child development. Families First intervention would comprise 12 group sessions. When the Families First group sessions ended, the children were usually nine- or 10- months old. | The group is to meet in a calm and child-friendly  environment, usually in the evening to enable  parents to participate after work (7). | Public health nurse qualification, group leaders are trained | In contrast  to Parents First, which targeted and focused on  working with parents/mothers while the children  were invited to engage in other play activities, the  focus of Families First became the whole family  (mother, father, and child). To accommodate the Finnish public healthcare system, the leaders in  Families First were employees of the municipality,  and integrating the intervention was a part of their  professional work. Therefore, a profession in mental  health, considered important in Parents First, was not required. Further, the two group leaders held  Families First sessions every other week, as opposed to one group leader holding weekly sessions in  Parents First. In addition to the group sessions, the Parents First format included individual consultations as well as educational hand-outs on  child development (7). |
| Tauriello et al. (2023) | | INSIGHT The Intervention Nurses Starting Infants Growing on Healthy Trajectories | To teach first-time mothers responsive parenting skills for the primary prevention of obesity compared with a safety control intervention. For example, mothers were taught how to respond sensitively. As children grew, mothers were also taught to establish developmentally appropriate limits and consistent routines (i.e., structure), elements of demandingness.  Human Ecology Theory (Bronfenbrenner, 1979 | Mothers and their newborns were recruited in-person by research staff shortly after delivery from one maternity ward.  Mothers were eligible to participate if they were primiparous, English-speaking and at least 20 years of age, and if their newborns were full-term (i.e. 37 weeks gestation), singleton, and had a birth weight of at least 2500 g  Mothers in the RP intervention received guidance from research nurses on how to respond to their child’s needs across 4 behavioral states: drowsy, sleeping, fussy, and alert (i.e. interactive play, feeding). Prior to the first study visit at infant age 3–4 weeks, mothers in the RP group were mailed intervention materials which included a video demonstrating infant soothing techniques, a CD with white noise tracks, and handouts on feeding guidance, lactation support, and postpartum depression. At study visits, mothers were provided with age-appropriate guidance in each behavioral domain. For example, feeding guidance focused on teaching mothers to recognize child hunger and satiety cues, age-appropriate portion sizes, and using food for hunger only (i.e., not as a reward, punishment or to soothe distress). | Face-to-face home visits and other material | Research nurses administered both the RP and control interventions at home visits (60-90-minute duration) at child age 3-4, 16, 28, and 40 weeks and at research centre visits at one and two years. | Child’s home | Research nurses | N/A |
| **Targeted parenting programmes/interventions** | | | | | | | | | |
| O’Farrelly et al. (2021) | Video-feedback Intervention (VIPP-SD) | | To promote positive parenting and sensitive discipline  Attachment Theory (Bowlby 1969), Social Learning Theory (Bandura 1961) | Recruitment to the screening stage was through face-to-face or postal contacts in health visiting services, supplemented by advertisements in other clinical and community services and online outlets. Caregivers whose children met eligibility owing to their SDQ score were contacted via telephone to determine the family’s full eligibility and interest in the trial.  Families were included if the parent(s) or caregiver(s) were older than 18 years and provided written informed consent and the child was aged 12 to 36 months and scored in the top 20% for externalizing behaviors on the SDQ.  During four core and two booster sessions, the intervener films parents interacting with their child (approximately 10 minutes) during play-based and challenging interactions and then provides focused feedback on the filmed interactions from the previous visit. | Face-to-face home-visits to families  Observation-based parent feedback session | Families in the VIPP-SD group were offered 6 home-based sessions of one- to two-hour duration every two weeks | Child’s home | Trained health professional predominantly public health nurses and nursery nurses, as well as a small number of professionals from therapy, psychology, and psychiatry backgrounds.  Interveners completed four days of VIPP-SD training and three supervised practice sessions. | The posttreatment assessment was changed from 4 to 5 months  after randomization early in the study, as the full intervention was not always completed within this time frame. |

References:

1. Dodge & Goodman. 2019. Universal reach at birth: Family Connects. The Future of Children 29(1), 41–60.

2. Nugent JK, Keefer CH, Minear S, Johnson LC & Blanchard Y. 2007. Understanding newborn behavior early relationships. Paul H. Brookes Publishing Co., Baltimore, Maryland.

3. The Regents of the University of Colorado. 2025. Nurse-Family Partnership. Accessed 29.3.2025. Available at https://nfpinternational.org/nfp-model/

4. Gov.UK. 2025. Family Nurse Partnership programme. Accessed 29.3.2025. Available at <https://www.gov.uk/guidance/family-nurse-partnership-programme>

5. Kliem S, Sandner M, Lohmann A, Sierau S, Dähne V, Klein AM & Jungmann T. 2018. Follow-up study regarding the medium term effectiveness of the home-visiting program “Pro Kind” at age 7 years: study protocol for a randomized controlled trial. Trials 19(323), 1–20.

6. Goldfeld S, Price A & Kemp L. 2018. Designing, testing, and implementing a sustainable nurse home visiting program: right@home. Annals of the New York Academy of Sciences 419, 141–159.

7. Kalland M, Fagerlund Å, von Koskull M & Pajulo M. 2016. Families First: the development of a new mentalization-based group intervention for first-time parents to promote child development and family health. Primary Health Care Research & Development 17, 3–17.
